# Pan-genomic analysis of bovine monocyte-derived macrophage gene expression in response to in vitro infection with *Mycobacterium avium* subspecies *paratuberculosis*

**DOI:** 10.1186/1297-9716-43-25

**Published:** 2012-03-28

**Authors:** David E MacHugh, Maria Taraktsoglou, Kate E Killick, Nicolas C Nalpas, John A Browne, Stephen DE Park, Karsten Hokamp, Eamonn Gormley, David A Magee

**Affiliations:** 1 Animal Genomics Laboratory, UCD School of Agriculture and Food Science, University College Dublin, Belfield, Dublin 4, Ireland; 2 UCD Conway Institute of Biomolecular and Biomedical Research, University College Dublin, Belfield, Dublin 4, Ireland; 3 Smurfit Institute of Genetics, Trinity College Dublin, Trinity College, Belfield, Dublin 2, Ireland; 4 Tuberculosis Diagnostics and Immunology Research Centre, UCD School of Veterinary Medicine, University College Dublin, Belfield, Dublin 4, Ireland

## Abstract

*Mycobacterium avium* subspecies *paratuberculosis* is the causative agent of Johne’s disease, an intestinal disease of ruminants with major economic consequences. Infectious bacilli are phagocytosed by host macrophages upon exposure where they persist, resulting in lengthy subclinical phases of infection that can lead to immunopathology and disease dissemination. Consequently, analysis of the macrophage transcriptome in response to *M. avium* subsp. *paratuberculosis* infection can provide valuable insights into the molecular mechanisms that underlie Johne’s disease. Here, we investigate pan-genomic gene expression in bovine monocyte-derived macrophages (MDM) purified from seven age-matched females, in response to in vitro infection with *M. avium* subsp. *paratuberculosis* (multiplicity of infection 2:1) at intervals of 2 hours, 6 hours and 24 hours post-infection (hpi). Differentially expressed genes were identified by comparing the transcriptomes of the infected MDM to the non-infected control MDM at each time point (adjusted *P*-value threshold ≤ 0.10). 1050 differentially expressed unique genes were identified 2 hpi, with 974 and 78 differentially expressed unique genes detected 6 and 24 hpi, respectively. Furthermore, in the infected MDM the number of upregulated genes exceeded the number of downregulated genes at each time point, with the fold-change in expression for the upregulated genes markedly higher than that for the downregulated genes. Inspection and systems biology analysis of the differentially expressed genes revealed an enrichment of genes involved in the inflammatory response, cell signalling pathways and apoptosis. The transcriptional changes associated with cellular signalling and the inflammatory response may reflect different immuno-modulatory mechanisms that underlie host-pathogen interactions during infection.

## Introduction

Johne’s disease in ruminants is caused by infection with the facultative intracellular pathogen *Mycobacterium avium* subspecies *paratuberculosis*―a mycobacterial species closely related to *M. tuberculosis* and *M. bovis*, the causative agents of human and bovine tuberculosis, respectively [1]. Johne’s disease is characterised by chronic granulomatous enteritis, persistent diarrhoea, progressive wasting and can result in death. However, *M. avium* subsp. *paratuberculosis* infection often remains asymptomatic with pathology largely restricted to the ileum, rendering diagnosis difficult [2,3]. Diagnosis is further confounded by the documented technical limitations of current diagnostic tests, which are based on faecal culture techniques, enzyme immunoassays that detect the presence of milk and serum antibodies directed against *M. avium* subsp. *paratuberculosis* antigens, and interferon-gamma (INF-γ) release assays [4,5].

*M. avium* subsp. *paratuberculosis* infection has major implications for domestic animal health worldwide causing significant economic loss in affected herds due to lost productivity and premature culling. In cattle, Johne’s disease results in estimated losses of $1.5 billion to the US dairy industry annually, while dairy herd prevalence of Johne’s disease is estimated ≥ 50% in certain US states and European countries [6-9]. Furthermore, *M. avium* subsp. *paratuberculosis* has been implicated as an agent that causes or exacerbates Crohn’s disease in humans; although this hypothesis remains highly contentious [10].

Exposure to *M. avium* subsp. *paratuberculosis* in ruminants generally occurs within the first months of life, through either a faecal–oral route or by ingestion of infected colostrum or milk, while some evidence suggests that infection can occur in utero [2,3,11,12]. Once internalised, infectious bacilli cross the intestinal mucosa by penetrating the M cells that cover the ileal Peyer’s patches. The bacilli are then phagocytosed by intestinal macrophages, which serve as key effector cells in initiating the appropriate innate and adaptive immune response necessary to determine the outcome of infection [13,14].

The immune response to *M. avium* subsp. *paratuberculosis* infection in ruminants is similar to that elicited by *M. tuberculosis* and *M. bovis* infection in humans and cattle, respectively [13]. Infected macrophages secrete several NF-κB-inducible inflammatory cytokines, such as tumour necrosis factor (TNF-α), interleukin 10 (IL-10) and interleukin 12 (IL-12), which initiate and regulate an adaptive immune response characterised by the release of IFN-γ from T-cells [15-17]. IFN-γ activates microbicidal activity in infected macrophages and also promotes the sequestration of the pathogen in granulomas―organised complexes of immune cells consisting of lymphocytes, non-infected macrophages and neutrophils that contain mycobacterial-infected macrophages and prevent the spread of bacilli to other tissues [1,13].

In many cases, however, the pathogen can evade the host immune response, resulting in its survival and propagation in infected macrophages within granulomas. This persistence can lead to a lengthy non-shedding subclinical phase of between 2–5 years during which *M. avium* subsp. *paratuberculosis* proliferates in the gut, ultimately resulting in the development of immunopathology that enables the dissemination of infection to other tissues and shedding from the host [18,19]. The survival of the pathogen in intestinal macrophages is believed to be achieved through a diverse range of mechanisms including the inhibition of phagosome ma-turation and the suppression of key immuno-regulatory pathways that mediate the host immune response to infection [20,21]. Therefore, analysis of the macrophage transcriptome in response to *M. avium* subsp. *paratuberculosis* infection may offer a deeper understanding of the cellular processes governing pathogen-macrophage interactions and how modulation of these cellular pathways can result in pathology. Furthermore, identification of transcriptional markers of infection may enable novel diagnostics for Johne’s disease, providing new tools for disease management.

On-going developments in mammalian genome resources and high-throughput genomic technologies continually provide improved methodologies for analysis of the gene expression changes induced in bovine macrophages and peripheral blood in response to *M. avium* subsp. *paratuberculosis* infection both in vivo and in vitro [17,22-25]*.* In the current study, we analyse genome-wide transcriptional changes in purified monocyte-derived macrophages (MDM) isolated from seven Holstein-Friesian females in response to *M. avium* subsp. *paratuberculosis* infection in vitro (multiplicity of infection (MOI) 2:1). Total cellular RNA was extracted from infected and non-infected control MDM from all seven animals at intervals of 0, 2, 6 and 24 hpi and prepared for global gene expression analyses using the pan-genomic high-density Affymetrix® GeneChip® Bovine Genome Array. Differentially expressed genes were identified through comparison of the transcriptional profiles from the infected and non-infected control MDM at each time point post-infection. Differentially expressed genes were further investigated using the Ingenuity® Systems Pathway Analysis (IPA) Knowledge Base in order to identify the macrophage cellular pathways underlying *M. avium* subsp. *paratuberculosis* infection. These data add a novel layer of information regarding the complex macrophage molecular pathways elicited upon *M. avium* subsp. *paratuberculosis* infection and the role these pathways play in establishing the host immune response to Johne’s disease.

## Material and methods

### Ethics statement

All animal procedures were carried out according to the provisions of the Cruelty to Animals Act (Irish Department of Health and Children licence number B100/3939) and ethical approval for the study was obtained from the UCD Animal Ethics Committee (protocol number AREC-P-07-25-MacHugh).

### Animals

Seven age-matched (four-year old) Holstein-Friesian females were used in the current study (animal numbers 700, 706, 713, 716, 721, 724R and 727R). All animals were maintained under uniform housing conditions and nutritional regimens at the UCD Lyons Research Farm (Newcastle, County Kildare, Ireland). The animals were sourced from a herd managed by the UCD Lyons Research Farm without a recent history of Johne’s disease. These cattle were also negative for infection with *Brucella abortus*, *M. bovis* (as confirmed by single intradermal tuberculin test), *Salmonella* Typhimurium, bovine herpesvirus 1 (BHV-1) and bovine viral diarrhoea (BVD) virus.

### Monocyte extraction and culture of bovine MDM

For monocyte isolation, 300 mL of whole blood was collected in acid citrate dextrose buffer (Sigma-Aldrich Ireland Ltd., Dublin, Ireland) in sterile bottles. Blood was layered onto Accuspin^TM^ tubes containing Histopaque® 1077 (Sigma-Aldrich Ireland Ltd., Dublin, Ireland) and following density gradient centrifugation, peripheral blood mononuclear cells (PBMC) were collected. Contaminating red blood cells (RBC) were removed following resuspension and subsequent incubation of the PBMC in RBC lysis buffer (10 mM KHCO_3_, 150 mM NH_4_Cl, 0.1 mM EDTA pH 8.0) for 5 min at room temperature. After incubation, PBMC were washed twice with sterile phosphate-buffered saline (PBS; Invitrogen^TM^, Life Technologies Corporation, Paisley, UK) before resuspending cells in PBS containing 1% bovine serum albumin (BSA; Sigma-Aldrich Ireland Ltd., Dublin, Ireland). Monocytes were then isolated using the MACS® protocol involving magnetically-charged MACS® MicroBeads conjugated to mouse anti-human CD14 antibodies (Miltenyi Biotec Ltd., Surrey, UK), which has been shown to be cross-reactive with bovine monocytes [26]. The MACS® protocol involves the incubation of cells with antibody-conjugated MACS® MicroBeads that target cell-specific antigenic markers (the monocyte CD14 cell surface marker was targeted by the MACS® protocol used in the current study). The sample-MicroBead mixture is then applied to a MACS® column placed in a magnetic MACS® separator. Cells lacking the CD14 marker pass through the column while the magnetically-labelled cells carrying the CD14 marker are retained within the column. The column is washed, removed from the separator and the CD14-carrying monocytes are subsequently eluted from the column and used for MDM purification and culture. All MACS® procedures were carried out according to the ma-nufacturers’ instructions.

The identity and purity of monocytes was confirmed by flow cytometry using an anti-CD14 fluorescein-labelled antibody (data not shown). This method has been previously shown by us to yield a purity of CD14^+^ cells ≥ 99% [27]. Purified monocytes were seeded at 1 × 10^6^/well in 24-well tissue culture plates using RPMI 1640 medium (Invitrogen^TM^, Life Technologies Corporation, Paisley, UK) containing 15% heat inactivated foetal calf serum (FCS; Sigma-Aldrich Ireland Ltd., Dublin, Ireland), 1% non-essential amino acids (NEAA; Sigma-Aldrich Ireland Ltd., Dublin, Ireland) and gentamicin (50 μg/mL; Sigma-Aldrich Ireland Ltd., Dublin, Ireland) and incubated at 37°C, 5% CO_2_. Following 24 hours incubation (day one) the media was replaced with 1 mL fresh antibiotic-containing media to remove any non-adhered cells. On day three, media was replaced with 1 mL antibiotic-free culture media (RPMI 1640 medium containing 15% heat inactivated FCS and 1% NEAA only). To ensure that the same number of MDM were subjected to different treatments between experiments, cells were dissociated on day five using 1× non-enzymatic cell dissociation solution (Sigma-Aldrich Ireland Ltd., Dublin, Ireland), counted and then re-seeded at 2 × 10^5^ cells/well in fresh 24-well tissue culture plates (Sarstedt Ltd., County Wexford, Ireland) using antibiotic-free culture media (RPMI 1640 medium containing 15% heat inactivated FCS and 1% NEAA). By day eight, 80-100% confluent monolayers of MDM were generated that displayed the characteristic macrophage morphology as confirmed by Giemsa staining (data not shown). Day eight MDM were used for the in vitro infection experiments with *M. avium* subsp. *paratuberculosis*.

### Culture of *M. avium* subsp. *paratuberculosis*

A single clinical isolate of *M. avium* subsp. *paratuberculosis* was provided by Mr Eamon Costello and Mr Kevin Kenny (the Irish Central Veterinary Research Laboratory, Backweston, County Kildare). The clinical isolate of *M. avium* subsp. *paratuberculosis* used was obtained from a field-infected animal sourced from a herd with confirmed Johne’s disease. This isolate was prepared on Herrold's Egg Yolk Agar supplemented with amphotericin, nalidixic acid, vancomycin, with mycobactin J (Becton, Dickinson Ltd., Oxford, UK). Colonies from the agar preparations were resuspended in sterile PBS, aliquoted (0.5 mL) and stored at -80°C until required for in vitro MDM infections. Preparation of *M. avium* subsp. *paratuberculosis* aliquots was performed in a Biosafety Containment Level 3 (CL3) laboratory and conformed to the national guidelines on the use of Hazard Group 3 infectious organisms. It is important to note however, that culturing of *M. avium* subsp. *paratuberculosis* may be performed in a CL2 laboratory.

### In vitro infection of bovine MDM with *M. avium* subsp. *paratuberculosis*

**Figure 1:**
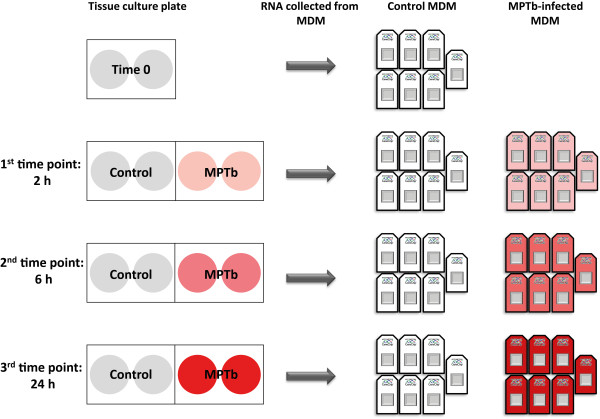
**Schematic depicting the experimental design used in the current study.** MDM were cultured in 24-well tissue culture plates and infected with *M. avium* subsp. *paratuberculosis* (MOI 2:1). RNA was extracted from infected (MPTb) and non-infected control MDM at three time points post-infection: 2 h, 6 h and 24 h. In addition, RNA was extracted from 0 h non-infected control MDM to assess potential non-experimental changes in gene expression. The MDM lysates from replicate tissue culture wells (shaded) were pooled for RNA extraction. Global gene expression for the non-infected control and infected MDM was analysed using the Affymetrix® GeneChip® Bovine Genome Array.

A schematic outlining the experimental design used in the current study is depicted in Figure 1. All infection experiments, including the preparation of non-infected control MDM, were performed in the CL3 laboratory (note: experimental infections involving *M. avium* subsp. *paratuberculosis* may be performed in a CL2 laboratory) [28]. Prior to the MDM infection experiments, MDM from two adjacent culture plate wells were lysed using RLT buffer from the RNeasy Mini kit (Qiagen Ltd., Crawley, UK) supplemented with 1% β-mercaptoethanol (Sigma-Aldrich Ireland Ltd., Dublin, Ireland) and pooled. These samples constituted the 0 h non-infected control MDM samples.

*M. avium* subsp. *paratuberculosis* aliquots (0.5 mL) were removed from −80°C storage and thawed for 30 min at room temperature prior to infection, placed in 9.5 mL RPMI 1640 medium (containing 15% heat inactivated FCS and 1% NEAA only), sterile filtered using a 5 micron filter (Millipore Ireland Ltd., County Cork, Ireland) to prevent clumping (the absence of clumps was confirmed by microscopy) and incubated at room temperature for 1 h before being counted using Petroff Hausser chamber (Fisher Scientific Ltd., Dublin, Ireland). MDM (seeded at 2 × 10^5^ cells/well) were infected with *M. avium* subsp. *paratuberculosis* (4 × 10^5^ cells/well) (multiplicity of infection 2:1) and incubated at 37°C, 5% CO_2_ for 2 h, 6 h and 24 h. For the non-infected control MDM samples at each time-point, antibiotic-free culture media (RPMI 1640 medium containing 15% heat inactivated FCS and 1% NEAA only) was added to each well. After 2 hpi, the media from all 6 h and 24 h infection experiments was replaced with 0.5 mL fresh antibiotic-free culture media per well and re-incubated at 37°C, 5% CO_2_ until MDM were required for harvesting. Infected and non-infected control MDM were lysed and harvested using RLT-1% β-mercaptoethanol buffer (Qiagen Ltd., Crawley, UK) at the designated time points. For each non-infected control and infected sample, MDM lysates from two culture plate wells were pooled and stored at -80°C until required for RNA extraction.

For colony-forming unit (cfu) counts, the *M. avium* subsp. *paratuberculosis* preparation used for the MDM infections was cultured using Middlebrook 7H11 medium (Difco^TM^, Becton, Dickinson Ltd., Oxford, UK) containing 0.50% (volume per volume) glycerol, 10% (volume/volume) Middlebrook (OADC) growth supplement (Becton, Dickinson Ltd., Oxford, UK) and 1 μg mycobactin J (Allied Monitor Inc., Fayette, MO, USA). Cfu counts yielded a mean MOI of 2:1.

### RNA extraction and microarray analysis

RNA was extracted using an RNeasy kit incorporating an on-column DNase treatment step (Qiagen Ltd., Crawley, UK) according to the manufacturer’s instructions. RNA quantity and quality was assessed using a NanoDrop^TM^ 1000 spectrophotometer (Thermo Fisher Scientific, Waltham, MA, USA) and an Agilent 2100 Bioanalyzer with an RNA 6000 Nano LabChip kit (Agilent Technologies Ltd., Cork, Ireland). All samples displayed a 260/280 ratio greater than 2.0 and RNA integrity numbers (RIN) greater than 8.5.

cDNA labelling, hybridisation and scanning for the microarray experiments were performed by Almac Diagnostics Ltd. (Craigavon, Co. Armagh, Northern Ireland) using a one-cycle amplification/labelling protocol on the Affymetrix GeneChip® Bovine Genome Array [29]. This array contains 24 072 probe sets representing over 23 000 gene transcripts and includes approximately 19 000 UniGene clusters. Technical replicates were not performed in this study. The procedures used for cDNA labelling, hybridisation and scanning for the microarray experiments are provided in the Additional Information. In total, 49 microarrays were prepared in the current study. These were generated from total RNA isolated from seven 0 h non-infected control MDM, seven 2 h non-infected control MDM, seven 2 h infected MDM, seven 6 h non-infected control MDM, seven 6 h infected MDM, seven 24 h non-infected control MDM and seven 24 h infected MDM.

### Statistical analysis of microarray data

Affymetrix® GeneChip® Bovine Genome Array data were analysed using the Bioconductor software project [30] contained within the R statistical environment [31]. Microarray quality control was performed using the Simpleaffy package contained within Bioconductor [32]. Normalisation of raw data was performed using the Factor Analysis for Robust Microarray Summarization (FARMS) algorithm within Bioconductor. The FARMS algorithm uses only perfect match probes and a quantile normalization procedure, providing both *P*-values and signal intensities [33]. Normalised data were then further subjected to filtering for informative probes sets using the Informative/Non-informative calls (I/NI-calls) [34] software package in R. This software package uses multiple probes for the same gene as repeated measures to quantify the signal-to-noise ratio of that specific probe set.

Differentially expressed genes were extracted using the Linear Models for Microarray Data (LIMMA) package [35] contained within Bioconductor. Genes displaying differential expression patterns between non-infected control and infected MDM were annotated using the Affymetrix® bovine gene annotation [36]. The Benjamini-Hochberg multiple testing correction method [37] was applied to all differentially expressed genes to minimise the false discovery rate (FDR) and adjusted *P*-values for differentially expressed genes were calculated. In this study, an adjusted *P*-value threshold of ≤ 0.10 was chosen.

Geometric mean fold-changes in gene expression are presented in the current study following back-transformation of mean log_2_ fold-changes obtained from statistical analysis of microarray data. For replicate probes for single genes, the average log_2_ expression fold-change was used and subsequently back-transformed. The negative reciprocals of the geometric fold-changes were calculated and are presented for downregulated genes.

All data are MIAME compliant [38] and have been submitted to the NCBI Gene Expression Omnibus (GEO) database [39] with experiment series accession numbers GSE35185. It is important to note that one microarray (6 h infected MDM RNA sample; animal number 713) failed quality control based on the analysis performed using the Simpleaffy package. This sample was omitted from the statistical analysis of the microarray data.

### Systems biology analyses

Ingenuity® Systems Pathway Analysis (IPA; Ingenuity Systems, Redwood City, CA, USA) was used to identify canonical pathways and functional processes of biological importance within the list of differentially expressed genes. The Ingenuity® Knowledge Base contains the largest database of manually-curated and experimentally-validated physical, transcriptional and enzymatic molecular interactions. Furthermore, each interaction in the Ingenuity® Knowledge Base is supported by previously published information.

For IPA analysis, the Affymetrix® GeneChip® Bovine Genome Array was used as a reference gene set. All differentially expressed genes with an adjusted *P* value ≤ 0.10 were included. For replicate probe IDs, the mean log_2_ expression fold-change was used. Only differentially expressed genes mapping to molecules in the Ingenuity® Knowledge Base were used for systems analysis. Functional analysis of genes was performed using IPA to characterise the biological functions of the differentially expressed genes between the non-infected control and infected MDM. For this, IPA performed an over-representation analysis that categorises the differentially expressed genes within the uploaded list into functional gene ontology (GO) categories using the Ingenuity® Knowledge Base. Each GO category in IPA is ranked based on the number of differentially expressed genes falling into each functional group. Right-tailed Fisher’s exact tests were used to calculate a *P*-value for each of the biological function assigned to list of differentially expressed genes.

IPA contains a large library of known canonical pathways that were overlaid with the differentially expressed genes to identify major biological pathways associated with *M. avium* subsp. *paratuberculosis* infection in MDM in vitro. The significance of the association between differentially expressed genes and the canonical pathway was assessed using two methods: (1) a ratio of the number of molecules from the differentially expressed gene data set that map to the pathway compared to the total number of molecules that map to the canonical pathway based on the reference gene list; and (2) a Fisher’s exact test that generates a *P*-value for the assignment of the differentially expressed genes to a particular canonical pathway compared to the reference gene list. Canonical pathways were then overlaid with the expression values of the differentially expressed genes.

### Conventional cDNA preparation for real time quantitative reverse transcription PCR (qRT-PCR) analysis

cDNA was prepared from 80 ng of total RNA (including the 0 h non-infected control RNA samples) using a High Capacity cDNA Reverse Transcription Kit (Applied Biosystems^TM^, Life Technologies Corporation, Warrington, UK) from all 49 RNA samples used in the microarray hybridisations. cDNA conversions were performed in 20 μL reactions using random primers as per the manufacturer’s instructions. In addition, 80 ng of pooled RNA (~10 RNA samples per pool) was included in non-reverse transcriptase (non-RT) control reactions to test for the presence of contaminating genomic DNA during real-time qRT-PCR analysis. All cDNA samples and non-RT controls were diluted 1:8 using RNAse- and DNAse-free water (yielding a final cDNA concentration of 0.5 ng/μL) and stored at −20°C prior to real time qRT-PCR analysis.

### Whole-transcriptome amplified cDNA preparation for real time quantitative reverse transcription PCR (qRT-PCR) analysis

To generate a repository of cDNA from each RNA sample, whole-transcriptome linearly amplified cDNA was prepared using the WT-Ovation^TM^ RNA Amplification System (NuGEN Technologies Inc., Bemmel, The Netherlands). For this, 25 ng of template RNA from all 49 RNA samples was included in each amplification reaction and all procedures were performed according to the manufacturer’s instructions. In addition, non-template controls that included RNAse- and DNAse-free water instead of RNA template were also prepared using the WT-Ovation^TM^ RNA Amplification System kit. Once amplification reactions were completed, the amplified cDNA and non-template controls were diluted 1:1000 using RNAse- and DNAse-free water and stored at −20°C prior to real time qRT-PCR analysis.

### Real time quantitative reverse transcription PCR (qRT-PCR) analysis and validation of microarray results

Intron-spanning primers were designed for each gene using the Primer3Plus package [40] and commercially synthesised (Eurofins MWG Operon, Ebersberg, Germany). Additional file 1: Table S1 provides experimental information for all primer pairs used. Real time qRT-PCR reactions (20 μL final volume) were performed on 96-well plates using Fast SYBR® Green Master mix (Applied Biosystems^TM^, Life Technologies Ltd., Warrington, UK) on a 7500 Fast Real-Time PCR System (Applied Biosystems^TM^, Life Technologies Corporation, Warrington, UK) as per manufacturer’s instructions. Real time qRT-PCR amplifications contained either: (a) 2 μL of the diluted conventionally-prepared cDNA/non-RT control (equivalent to 1.0 ng of total RNA template); or (b) 2 μL of the diluted linearly amplified cDNA/non-RNA template control. A final concentration of 300 nM of each forward and reverse primer was included in each amplification reaction. Non-template real time qRT-PCR controls, non-RT controls and a seven-point standard curve prepared from 1:2 serial dilutions of pooled conventionally-prepared cDNA or linearly amplified cDNA from each infected and non-infected control MDM sample were included on every real time qRT-PCR plate.

PCR thermal cycling conditions for each amplicon comprised one cycle at 50°C for 2 min, one cycle at 95°C for 20 s, followed by 40 cycles at 95°C for 3 s and 60°C for 30 s. A dissociation step was included for all amplifications to confirm the presence of single discrete PCR products of the expected size; this was further confirmed by visualisation of the amplification products on 2% agarose gels stained with 0.5 μg/mL ethidium bromide (Invitrogen^TM^, Life Technologies Corporation, Paisley, UK).

### Statistical analysis of real time qRT-PCR data

All real time qRT-PCR data, including estimation of primer efficiencies estimations using the standard curves, were analysed using the qbase^PLUS^ software package [41]. Real time qRT-PCR efficiency correction and normalisation was performed using the peptidylprolyl isomerase A (cyclophilin A) gene (*PPIA*) based on GeNorm analysis performed by us in a previous study involving bovine MDM [27]. *PPIA* expression profiles were generated for both the conventionally-prepared and linearly amplified cDNA templates for all samples to ensure appropriate real time qRT-PCR efficiency correction and normalisation analyses were performed for all genes-of-interest.

Calibrated normalised relative quantities (CNRQ) of gene expression for each analysed sample as generated by the qbase^PLUS^ package were used to calculate fold-changes in expression for each gene. For this, the CNRQ value generated for each infected MDM sample was divided by the CNRQ value generated for the non-infected control MDM sample at the corresponding post-infection time point from the same animal. Log_2_ fold-changes in gene expression were then used for the statistical analysis of all genes analysed using real time qRT-PCR. All statistical analyses were performed using the SPSS version 18 (IBM Corporation, Armonk, NY, USA) and Minitab version 16 (Minitab Ltd., Coventry, UK) statistical packages.

Anderson-Darling tests of normality were applied to the residuals of the log_2_ fold-change values at each time point prior to statistical analysis to ensure the data conformed to a normal distribution–no significant departures from normality were observed for any of the genes analysed (*P* ≥ 0.05). Two-tailed paired Student’s *t*-tests were used to assess significant differences in mean log_2_ fold-changes in gene expression between the infected MDM and the non-infected control MDM at each time point post-infection. Geometric mean fold-changes in expression were generated and are presented for each gene by back-transformation of the log_2_ fold-change data. The negative reciprocals of the geometric fold-changes were calculated and are presented for downregulated genes.

To validate the performance of the linearly amplified cDNA in real time qRT-PCR amplifications and to ensure that no over- or under-representation of mRNA transcripts was introduced into the samples following amplification, the expression profiles of three genes (*CCL5*, *CCL20* and *IL1B*) were generated using both conventionally prepared cDNA and linearly amplified cDNA. The log_2_ fold-changes in expression in the infected MDM relative to the non-infected control MDM at each time point were then compared for both template types using regression analysis.

## Results

### Analysis of gene expression 2 hpi

**Figure 2:**
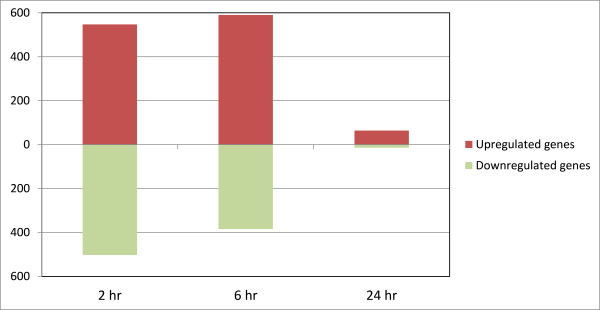
**The number of significantly differentially expressed genes at each time point post-infection with* M. avium *subsp. *paratuberculosis*.** The number of upregulated and downregulated genes in the infected MDM relative to the non-infected control MDM at each time point are shown (adjusted *P*-value ≤ 0.10).

A total of 1232 transcripts, representing 1050 unique genes, were identified as being differentially expressed between the infected and non-infected control MDM samples 2 hpi (adjusted *P*-value ≤ 0.10). Of these, 673 transcripts (representing 547 unique genes) were upregulated with the remaining 559 transcripts (representing 503 unique genes) displaying downregulation in the infected MDM (Figure 2). In addition, the relative fold-change in expression was markedly higher for the upregulated genes compared to the downregulated genes at this time point. A complete list of the differentially expressed genes and the relative fold-change in expression at the 2 h time point post-infection are provided in Additional file 2: Table S2.

Further inspection of the differentially expressed genes revealed that many of the highly upregulated genes at this time point had immune-related functions, particularly NF-κB-inducible chemokine and cytokine genes, such as the chemokine (C-X-C motif) ligand 2 gene (*CXCL2*); the interleukin 1, beta gene (*IL1B*); the interleukin 1, alpha gene (*IL1A*); the tumour necrosis factor gene (*TNF*); the chemokine (C-C motif) ligand 20 gene (*CCL20*) and the interleukin 6 and interleukin 10 genes (*IL6* and *IL10*). The gene encoding nitric oxide synthase 2 (*NOS2*), whose function is to produce reactive nitric oxide―a potent effector molecule involved in the regulation of the host immune response to mycobacterial infection [42]―was also upregulated 2 hpi. Finally, the genes encoding the NF-κB1 (*NFKB1*) and NF-κB2 (*NFKB2*) members of the NF-κB transcription factor complex were upregulated in the infected MDM at this time encoding proteins belonging to the mitogen-activated protein kinase (MAPK) signalling cascade―which is involved in the activation of downstream cellular responses upon the recognition of mycobacterial pathogen-associated molecular patterns (PAMPs) by cell surface pathogen recognition receptors (PRRs), such as the Toll-like receptors (TLRs) and the receptor tyrosine kinases (RTKs) [15]―were also differentially expressed at this time point. These included members of the mitogen-activated protein kinase kinase kinases (MAPKKKs), the mitogen-activated protein kinase kinases (MAPKKs) and the mitogen-activated protein kinases (MAPKs). Notably, genes encoding MAPK-activated transcription factors, such as the JUN proto-oncogene (*JUN*) and the FBJ murine osteosarcoma viral oncogene homolog gene (*FOS*), displayed the greatest reduction in fold-change expression in the infected samples.

The total number of differentially expressed genes 2 hpi that could be mapped to molecules in the Ingenuity® Knowledge Base was 841 from a total of 1232 differentially expressed transcripts. Functional categorisation analysis using IPA showed that the differentially expressed genes 2 hpi had roles involved in the regulation of gene transcription, the immune response, cell signalling, and cell death ( Additional file 3: Table S3). IPA was also used to identify the top ranking canonical pathways enriched for differentially expressed genes in response to *M. avium* subsp. *paratuberculosis* infection. Many of the top ten ranking canonical pathways identified had an immunological role and included *Interleukin 10 signalling* (1^st^ ranked pathway), *Dendritic cell maturation* (2^nd^ ranked pathway), *TNFR2 signalling* (3^rd^ ranked pathway), *The role of macrophages in rheumatoid arthritis* (4^th^ ranked pathway) and *B cell activating factor signalling* (5^th^ ranked pathway) ( Additional file 4: Table S4).

### Analysis of gene expression 6 hpi

Comparison of the microarray expression profiles of the infected MDM and non-infected control MDM 6 hpi revealed a total of 1137 differentially expressed transcripts, representing 974 unique genes (adjusted *P*-value ≤ 0.10). Of these, 713 transcripts (representing 590 unique genes) were upregulated with the remaining 424 transcripts (representing 384 unique genes) displaying downregulation in the infected MDM relative to the non-infected control MDM (Figure 2). As with the data from the 2 hpi time point, the relative fold-change in expression was markedly higher for the upregulated genes compared to the downregulated genes at this time point. A complete list of the differentially expressed genes and the relative fold-change in expression at the 6 hour time point post-infection are provided in Additional file 5: Table S5.

Immune-related genes were among the differentially expressed genes showing the highest relative increase in expression; however, the fold-change induction of these genes was not as high as those detected at the 2 h time point. These genes included the chemokine (C-C motif) ligand 4, 5 and 20 genes (*CCL4*, *CCL5* and *CCL20*); the CD40 molecule, TNF receptor superfamily member 5 gene (*CD40*), and the complement factor B gene (*CFB*). *CXCL2*, *IL1B*, *NFKB1*, *NFKB2*, *NOS2* and *TNF* were also upregulated at this time point. *IL10* was not differentially expressed at this time point, while the genes belonging to the MAPK signalling pathway that were identified as being differentially expressed at the 2 hpi time point were not differentially expressed at 6 hpi.

The total number of differentially expressed genes that could be mapped to molecules in the Ingenuity® Knowledge Base 6 hpi was 768 from a total of 1137 differentially expressed transcripts. Functional categorisation of the differentially expressed genes 6 hpi revealed enrichment for genes involved in the regulation of cellular deve-lopment, inflammation, adaptive immune mechanisms and cell death ( Additional file 6: Table S6). The top ranking IPA-identified canonical pathways enriched for differentially expressed genes in response to *M. avium* subsp. *paratuberculosis* infection are detailed in Additional file 7: Table S7. Among the top five ranking canonical pathways had an immunological role and included *TREM1 signalling* (1^st^ ranked pathway); *B cell receptor signalling* (2^nd^ ranked pathway); *Altered T cell and B cell signalling in rheumatoid arthritis* (3^rd^ ranked pathway); and *Communication between innate and adaptive immune cells* (5^th^ ranked pathway).

Notably, inspection of the differentially expressed genes within the 4^th^ ranked canonical pathway, *the role of pattern recognition receptors in the recognition of bacteria and viruses*, showed that the genes encoding TLR3 and the interferon induced with helicase C domain 1 (IFIH1) proteins were upregulated following infection. This is of note, as TLR3 and IFIH1 are intracellular PRRs largely involved in the recognition of viral PAMPs, such as double-stranded reoviral RNA.

### Analysis of gene expression 24 hpi

A total of 86 transcripts, representing 78 unique genes, were identified as being differentially expressed between the infected and non-infected control MDM 24 hpi (adjusted *P*-value ≤ 0.10). Of these, 72 transcripts (representing 64 unique genes) were upregulated with the remaining 14 transcripts (representing 14 unique genes) displaying downregulation in the *M. avium* subsp. *paratuberculosis*-infected MDM (Figure 2). As with the 2 h and 6 h time points post-infection, the relative fold-change in expression was higher for the upregulated genes compared to the downregulated genes at this time point. However, the fold-change in expression for the upregulated genes at the 24 h time point was substantially lower than that for the upregulated genes at the earlier time points. A complete list of the differentially expressed genes and the relative fold-change in expression at the 24 h time point post-infection are provided in Additional file 8: Table S8.

Several of the differentially expressed genes identified at the 24 hpi time point had no obvious immunological role. Of the differentially expressed genes that had a known immune function were the serum amyloid A3 gene (*SAA3*); the complement factor B gene (*CFB*); a small number of members of the C-type lectin/C-type lectin-like domain protein superfamily, such as the genes encoding the C-type lectin domain family 4, member E (*CLEC4E*) and C-type lectin domain family 2, member D proteins (*CLEC2D*); and *CD40*―all of these genes were upregulated at this time point. Although we analysed the list of 78 differentially expressed genes using IPA (59 differentially expressed genes from 86 differentially expressed transcripts mapped to molecules in IPA at this time point), these proved largely uninformative given the low number of differentially expressed genes represented within each functional category and canonical pathway ( Additional file 9: Tables S9 and Additional file 10: S10).

### Real time quantitative reverse transcription PCR (qRT-PCR) analysis and validation of microarray results

A panel of the 15 genes identified through analysis of the microarray data and previous in vitro mycobacterial infection studies conducted in our laboratory [43] were selected for real time qRT-PCR analysis and microarray validation. Selection of these genes was based largely on the highest fold increase or decrease in gene expression following infection with *M. avium* subsp. *paratuberculosis* and/or lowest adjusted *P*-value, using data primarily from the 2 h and 6 h time points; however, gene selection also included information from the 24 h time point where possible. The genes selected for real time qRT-PCR analysis were: *AREGB, CCL4CCL5CCL20CD40CFBCXCL2IL1BIL6IL15IRF1NFKB2PIK3IP1SPRY2* and *TNF*. cDNA prepared from all 49 RNA samples were included for real time qRT-PCR analysis.

Of the 15 genes analysed using real time qRT-PCR, nine genes (*CCL4*, *CD40*, *CFB*, *CXCL2*, *IL6*, *IL15*, *IRF1*, *NFKB2* and *TNF*) were analysed using conventionally-prepared cDNA template only; three genes (*CCL5*, *CCL20* and *IL1B*) were analysed using both the conventionally-prepared cDNA and the linearly amplified cDNA templates in order to validate the performance of the linearly amplified cDNA; while the remaining three genes (*AREGB*, *PIK3IP1* and *SPRY*) were analysed using the linearly amplified cDNA template only.

Firstly, to validate the performance of the linearly amplified cDNA in real time qRT-PCR amplifications, the expression profiles generated for the *CCL5*, *CCL20* and *IL1B* genes using conventionally-prepared and linearly amplified cDNA were compared. For this, regression analysis was performed using the log_2_ fold-changes in relative gene expression for the infected MDM analysed for the two different cDNA templates across all three time points, revealing a high correlation for all three genes (*r*^*2*^ ≥ 0.82, *P* ≤ 0.001) (Figure S1). Furthermore, the mean-fold changes in expression (and their associated *P*-values) in the infected MDM relative to the non-infected control MDM for these genes were similar across all three time points ( Additional file 12: Figure S1 and Additional file 11: Table S11). Only *IL1B* was shown to be significantly differentially expressed at 24 hpi using the conventionally prepared cDNA, while this gene was not differentially expressed at this time point based on data from the linearly amplified cDNA. These results suggest that no significant preferential amplification bias of gene transcripts or amplification artefacts occurred during the preparation of the linearly amplified cDNA. Consequently, we concluded that the use of the linearly amplified cDNA was suitable in the current study.

**Figure 3:**
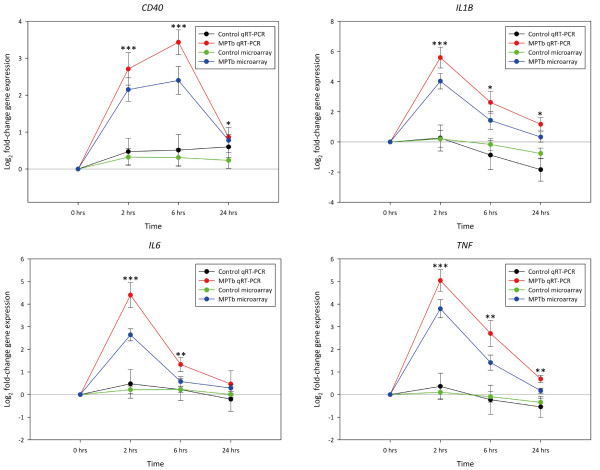
**Real time qRT-PCR analysis of the* CD40, IL1B, IL6 and TNF* genes following infection with *M. avium *subsp.* paratuberculosis*.** Log_2_ fold-changes in expression in the *M. avium* subsp. *paratuberculosis*-infected MDM (MPTb) relative to the non-infected control MDM at all three time points are shown. All data presented is based on the conventionally-prepared cDNA template. For comparison, the expression profiles for these genes as per the microarray data are also shown. The significance of the fold-changes in expression for each gene based on the real time qRT-PCR analysis only are denoted by asterisks in the figure (*P ≤ 0.05, **P ≤ 0.01, ***P ≤ 0.001). In addition, the log_2_ fold-change in expression for the non-infected control MDM at each time point relative to the 0 hour non-infected control MDM are also shown for both the microarray and real time qRT-PCR data; no significant differences in gene expression between the non-infected control MDM relative to the 0 h non-infected control was observed at each time point (*P* ≥ 0.05).

The relative fold-changes in expression between the infected and non-infected control MDM at each time point post-infection for the *CD40IL1BIL6* and *TNF* genes as determined through real time qRT-PCR analysis are depicted graphically in Figure 3, while data for all other genes analysed through real time qRT-PCR are presented in Additional file 13: Figure S2. Mean fold-changes in gene expression are presented in Table 1, as are descriptions of the function of each gene, which were obtained from the Entrez Gene [44] and the GeneCards version 3 databases [45]. Fourteen of the 15 genes analysed using real time qRT-PCR were significantly differentially expressed at the 2 h time point, while 13 and seven genes were significantly differentially expressed at the 6 h and 24 h time points, respectively (*P* ≤ 0.05). In total, six genes (*CCL5CD40CFBIL1BIRF1* and *TNF*) were significant upregulated across all three time points (*P* ≤ 0.05), while only one gene (*AREGB*) was significantly downregulated in the infected MDM samples across all time points (*P* ≤ 0.05). Furthermore, comparison of the expression profiles of each gene for the 2 h, 6 h and 24 h non-infected control MDM relative to the 0 h non-infected control MDM revealed significant differentially expression for only the *AREGB* gene at the 2 h and 6 h time points (*P* ≤ 0.01). The expression profiles of the 2, 6 and 24 h non-infected control MDM for the remaining 14 genes were not significantly different relative to the 0 h non-infected control MDM (*P* ≥ 0.05).

**Table 1:** Comparison of fold-changes in gene expression in the infected MDM relative to the non-infected control MDM based on microarray and real time qRT-PCR results

**Gene symbol**	**Gene name**	**Gene description**	**2 h mean fold-changes in gene expression**			**6 h mean fold-changes in gene expression**			**24 h mean fold-changes in gene expression**		
			**Microarray data**	**Real time qRT-PCR**	***P*-value (real time qRT-PCR)**	**Microarray data**	**Real time qRT-PCR**	***P*-value (real time qRT-PCR)**	**Microarray data**	**Real time qRT-PCR**	***P*-value (real time qRT-PCR)**
*AREGB*	Amphiregulin B	A growth-modulating glycoprotein	NS	- 1.29	≤ 0.01	- 2.03	- 2.58	≤ 0.05	NS	- 1.54	≤ 0.01
*CCL4*	Chemokine (C-C motif) ligand 4	A proinflammatory and chemotactic chemokine	+ 6.59	+ 28.87	≤ 0.001	+ 3.11	+ 9.67	≤ 0.05	NS	+ 1.28	NS
*CCL5*	Chemokine (C-C motif) ligand 5	A proinflammatory chemokine involved in the chemotaxis of monocytes and T-helper cells	+ 3.56	+ 5.07	≤ 0.05	+ 4.02	+ 4.60	≤ 0.05	NS	+ 2.11	≤ 0.05
*CCL20*	Chemokine (C-C motif) ligand 20	A chemokine involved in the chemoattraction of lymphocytes and neutrophils	+ 10.47	+ 63.19	≤ 0.001	+ 2.80	+ 10.82	≤ 0.05	NS	+ 1.22	NS
*CD40*	CD40 molecule, TNF receptor superfamily member 5	A member of the TNF-receptor superfamily. Mediates the immune and inflammatory responses	+ 4.44	+ 6.55	≤ 0.001	+ 5.23	+ 10.80	≤ 0.001	+ 1.71	+ 1.82	≤ 0.05
*CFB*	Complement factor B	A component of the alternative pathway of complement activation	+ 3.54	+ 4.61	≤ 0.05	+ 4.84	+ 8.64	≤ 0.01	+ 2.98	+ 4.19	≤ 0.001
*CXCL2*	Chemokine (C-X-C motif) ligand 2	An immunoregulatory chemokine produced by activated monocytes and neutrophils at sites of inflammation	+ 14.34	+ 28.56	≤ 0.001	+ 2.38	+ 3.07	≤ 0.01	NS	+ 1.45	NS
*IL1B*	Interleukin 1, beta	A cytokine that mediates the inflammatory response including cell proliferation, differentiation and apoptosis	+ 14.41	+ 48.53	≤ 0.001	+ 2.97	+ 6.16	≤ 0.05	NS	+ 2.25	≤ 0.05
*IL6*	Interleukin 6	A cytokine that functions in inflammation and the maturation of B cells	+ 6.25	+ 21.12	≤ 0.001	NS	+ 2.51	≤ 0.01	NS	+ 1.38	NS
*IL15*	Interleukin 15	A cytokine that regulates T and natural killer cell activation and proliferation	+ 3.51	+ 4.40	≤ 0.001	+ 2.03	+ 2.73	≤ 0.01	NS	1.04	NS
*IRF1*	Interferon regulatory factor 1	A member of the interferon regulatory transcription factor family. An activator of interferon alpha and beta transcription	+ 6.57	+ 12.64	≤ 0.001	+ 3.60	+ 5.20	≤ 0.01	NS	+ 1.60	≤ 0.01
*NFKB2*	Nuclear factor of kappa light polypeptide gene enhancer in B-cells 2 (p49/p100)	A pleiotropic transcription factor involved in inflammation, immunity, differentiation, cell growth and apoptosis	+ 3.41	+ 5.37	≤ 0.001	+ 3.69	+ 5.82	≤ 0.001	NS	+ 1.34	NS
*PIK3IP1*	Phosphoinositide-3-kinase interacting protein 1	Suppresses the activity of phosphatidylinositol-3-kinase (PI3K), a regulator of cell division	- 1.70	- 2.34	≤ 0.05	NS	- 1.36	NS	NS	+ 1.54	NS
*SPRY2*	Sprouty homolog 2 (*Drosophila*)	An inhibitor of receptor tyrosine kinase signalling proteins	- 2.07	- 2.20	NS	NS	- 1.12	NS	NS	- 1.46	NS
*TNF*	Tumor necrosis factor (TNF superfamily, member 2)	A proinflammatory cytokine (secreted by macrophages) involved in the regulation cell proliferation, differentiation and apoptosis, and coagulation.	+ 13.89	+ 33.01	≤ 0.001	+ 2.73	+ 6.51	≤ 0.01	NS	+ 1.61	≤ 0.01

Geometric mean fold-changes of gene expression (infected MDM relative to non-infected control MDM from the same animal at each time point) are given for the microarray and real time qRT-PCR data at each time point. “NS” indicates that the gene was not significantly differentially expressed at the appropriate time point. The *P*-values obtained from analysis of the real time qRT-PCR analysis are presented. Geometric fold-changes given for the microarray data were significantly differentially expressed using an adjusted *P*-value ≤ 0.10. Descriptions of the function of each gene were obtained from the Entrez Gene database [44] and the GeneCards version 3 database [45].

Comparisons between the results from the real time qRT-PCR and microarray analyses for all 15 genes are given in Table 1. In general, gene expression profiles for all 15 genes analysed with real time qRT-PCR were the same as those detected based on microarray analysis across all three time points. The expression profiles of *AREGB* and *SPRY2* at the 2 h time point, *IL6* at the 6 h time point and *AREGB*, *CCL5*, *IL1B*, *IRF1* and *TNF* at the 24 h point were different between the two datasets. Of the total 45 pairwise comparisons between the microarray data and the real time qRT-PCR data (i.e. 15 genes × 3 time points), eight were discordant, giving a concordance rate between the two datasets of 82% (i.e. 37/45).

The observed discrepancies between the microarray and real time qRT-PCR data for these genes may reflect differences in the sensitivity of the two analytical methods used and/or differences in the mRNA transcripts targeted by the probes (microarray) and primer pairs (real time qRT-PCR) used for the two forms of gene expression analysis [46]. In addition, the differences between the two datasets may reflect the application of the Benjamini-Hochberg multiple testing correction method [37] to all differentially expressed genes identified through the analysis of the microarray data (adjusted *P*-value threshold ≤ 0.10). This statistical correction was not applied to the real time qRT-PCR as the genes selected for this analysis were based on: (1) the list of differentially expressed genes identified from the microarray data presented in the current study, and (2) data generated by us from previous studies investigating the bovine immune response to in vivo and in vitro mycobacterial infections [43]. Therefore, analysis of the real time qRT-PCR analysis data generated in the current study was based on a priori knowledge and was not subject to the same rigorous post-hoc statistical correction as the microarray data [47-49].

## Discussion

High-throughput transcriptomic technologies, coupled with continually improving genome resources, have greatly enhanced the ability to interrogate global changes in gene expression underlying the cellular response to mycobacterial infection [23,24,50-53]. More specifically, these studies have enabled a deeper knowledge of the complex interactions between host mammalian macrophages and mycobacterial pathogens in vitro, thus, providing a model for understanding the molecular basis of mycobacterial disease. However, most of this research has focussed on the host transcriptional response to *M. tuberculosis* and *M. bovis* infection in humans and cattle, respectively, with a paucity of studies centred on the host immune response to *M. avium* subsp. *paratuberculosis* infection. Consequently, we have investigated transcriptional changes in bovine MDM isolated from seven Holstein-Friesian females in response to *M. avium* subsp. *paratuberculosis* infection in vitro using the genome-wide high-density Affymetrix® GeneChip® Bovine Genome Array at 2, 6 and 24 hpi. The gene expression data from these analyses were used to investigate key host macrophage signalling pathways involved during early *M. avium* subsp. *paratuberculosis* infection.

Comparison of the gene expression profiles in the infected MDM relative to the non-infected control MDM at each time point revealed a sequential decrease in the number of differentially expressed genes across the three time points analysed. In particular, there was a dramatic reduction in the number of differentially expressed genes at the 24 h time point (*n* = 78) compared to the number of differentially expressed genes detected at the 2 h (*n* = 1050) and 6 h (*n* = 974) time points, respectively. These results indicate that the majority of transcriptional changes occur within the first 6 hpi, with differential gene expression having largely abated at the 24 h time point. In addition, the number of upregulated genes exceeded the number of downregulated genes at each time point (547 upregulated/503 downregulated at 2 h; 590 upregulated/384 downregulated at 6 h; 64 upregulated/14 downregulated at 24 h), while the relative fold-change in expression for the upregulated genes was greater than that for the downregulated genes at each time point. These results are similar to previous findings demonstrating an over-representation of upregulated genes in peripheral blood mononuclear cells (PBMC) isolated from Johne’s disease-positive cattle following in vitro stimulation with *M. avium* subsp. *paratuberculosis*, while the number of differentially expressed genes also decreased dramatically across a 16 h post-stimulation time course [54].

Previous functional genomic studies have shown that in vitro and in vivo models of tuberculosis infection are associated with repression of host gene expression [51,55-57]―a strategy that is proposed to enable mycobacterial persistence in the host [58,59]. Furthermore, gene expression data generated from a recent in vitro *M. bovis*-bovine MDM infection study performed by our group (MOI 2:1) revealed that the number of differentially expressed genes increased sequentially across 2, 6 and 24 h intervals post-infection [43]. In addition, the number of downregulated genes in the *M. bovis*-infected MDM exceeded the number of upregulated genes at each time point, thus lending further support to the hypothesis that *M. bovis* infection is association with transcriptional repression [43].

The increased number of upregulated genes compared with downregulated genes, coupled with the reduction in the number of differentially expressed genes at each time point post-infection, as observed in the current study, supports previous work showing that *M. avium* subsp. *paratuberculosis* infection causes transient upregulation of host gene expression [54,60]. Furthermore, differences in the number and expression profiles (*i.e.* up- or downregulation) of MDM genes expressed following *M. avium* subsp. *paratuberculosis* and *M. bovis* infection in vitro, as observed here and by us previously [43], indicate that different macrophage responses are elicited upon infection with different mycobacterial species. These results may also highlight different immuno-modulatory strategies used by different mycobacterial pathogens to survive and prolife-rate in host macrophages.

Inspection and systems analysis of the list of differentially expressed genes identified following *M. avium* subsp. *paratuberculosis* infection in the present study enabled further understanding of the major cellular mechanisms occurring at each time point post-infection. For example, *TLR2* was the only TLR-encoding gene found to be differentially expressed (upregulated) in the infected MDM at the 2 h time point; a result that suggests that this PRR plays an important role in the early macrophage recognition of *M. avium* subsp. *paratuberculosis*. This finding supports the conclusions of previous studies which demonstrate that *TLR2* expression is upregulated in bovine monocytes following *M. avium* subsp. *paratuberculosis* infection in vitro [61] and also in subclinical and clinical forms of sheep paratuberculosis [62].

NF-κB-inducible cytokine and chemokine genes (such as *CCL20CXCL2IL1BIL1AIL6* and *TNF*), whose expression profiles are known to be altered following mycobacterial recognition by TLR2 [63,64], displayed the highest fold-increase in relative expression following *M. avium* subsp. *paratuberculosis* infection at the 2 h time point. These findings confirm that endogenous macrophage cytokine and chemokine gene expression is a key early event during mycobacterial infection in vitro [27,50,65-67]. Notably, the gene encoding the TLR2 adaptor protein, myeloid differentiation primary response 88 (*MYD88*), and the genes encoding other downstream TLR-signalling proteins, such as the interleukin-1 receptor-associated kinases (IRAKs), were not differentially expressed at this time point. Thus, it is possible that *M. avium* subsp. *paratuberculosis* infection activates TLR2-mediated signalling via a non-canonical pathway involving less well-defined TLR signalling pathway protein adaptors and intermediates [68]. However, as this is a transcriptomics study, the functional role of various pre-existing TLR adaptor proteins in macrophage cellular pathways during *M. avium* subsp. *paratuberculosis* infection cannot be assessed at the RNA level and consequently, their involvement in these pathways cannot be fully excluded. Further work involving both transcriptomic and proteomic platforms is required to investigate fully a role for macrophage non-canonical TLR signalling pathways in response to *M. avium* subsp. *paratuberculosis* infection.

Genes encoding members of the MAPK signalling cascade were also differentially expressed at the 2 h time point post-infection. The MAPK signalling cascade consists of evolutionarily conserved serine/threonine kinases that relay extracellular stimuli (often mediated through activation of TLRs [69]) to transcription factors that induce the expression of genes whose encoded products regulate various cellular processes including the innate immune responses. The MAPK signalling cascade is comprised of a three-tiered kinase system in which MAPKs―such as the p38 MAPK isoforms (p38α, β, γ and δ), the c-JUN N-terminal kinases (JNK1, 2 and 3) and the extracellular related kinases (MAPK-ERKs 1,2 and 3)―are activated through phosphorylation by MAPKKs (also called MAP2K/MKK/MEK proteins), which are activated through phosphorylation by MAPKKKs (also called MAP3K/MEKK proteins). Upon activation, MAPKs can translocate to the nucleus where they phosphorylate and activate several nuclear targets, including the FOS, JUN, and SP1 transcription factors that regulate the expression of genes encoding inflammatory chemokine and cytokines, including IL-1, IL-10, IL-12 and TNF-α [70,71].

In the current study, genes encoding members of the three tiers of the MAPK signalling cascade were differentially expressed in the infected MDM 2 hpi, some of which displayed opposing expression profiles. For example, *MAP3K14* (also called *NIK*) was downregulated, while *MAP3K8* was upregulated; *MAP2K3* and *MAP2K4* were both upregulated; *MAPK6* (also called ERK3) was upregulated and *MAPK14* (also called MAPK-p38α) was downregulated at this time point. In addition, *FOS* and *JUN* were downregulated at this time point post-infection.

These data support results from previous studies which demonstrate that MAPK signalling is modulated upon infection, a mechanism that may enable mycobacterial persistence in host macrophages [58]. For example, chemical inhibition of MAP2K1 (also called MEK1) and MAPK-ERK activity resulted in reduced TNF-α secretion and enhanced replication of pathogenic *M. avium* in infected human MDM in vitro [72]. Furthermore, enhanced phosphorylation of MAPK14 has been shown to be primarily responsible for upregulation of *IL10*, which encodes a key mediator of the host innate immune response to mycobacterial infection [73,74]. IL-10 is an immuno-regulatory cytokine produced by macrophages and other immune cells (such as T and B lymphocytes) that suppresses anti-mycobacterial activity―possibly by inhibiting NF-κB activity in target cells [75]―thus, limiting the level of local cytokine-induced tissue damage and systemic inflammatory responses during infection [76]. Interestingly, Weiss et al. [61] demonstrated that inhibition of IL-10 production in human monocytes following *M. avium* subsp. *paratuberculosis* infection in vitro result in enhanced phagosome matu-ration and mycobactericidal mechanisms. These findings have led to the proposal that mycobacteria modulate MAPK signalling pathways―particularly by inducing increased phosphorylation of MAPK14―resulting in the upregulation of *IL10* causing subsequent suppression of host innate immune responses to infection and enhanced mycobacterial intracellular proliferation [15,77].

In the current study, *IL10* was upregulated 2 hpi while *IL-10 signalling* was the top ranking canonical pathway identified at this time point. These results confirm the important role of IL-10 during *M. avium* subsp. *paratuberculosis* infection and support the hypothesis that mycobacteria exploit the anti-inflammatory activity of this cytokine to promote intracellular survival. However, the downregulation of *MAPK14* suggest that the induction of *IL10*, as observed here, does not involve increased *MAPK14* expression; instead *IL10* upregulation may be due post-transcriptional modification of MAPK14 (e.g. phosphorylation) not detected here or may be induced through an alternative cellular signalling mechanism.

Previous investigations have highlighted the important role of apoptosis in the host response to mycobacterial infection. In particular, macrophage apoptosis is increasingly regarded as host innate immune mechanism controlling mycobacterial infection by containing and limiting mycobacterial growth [78]. In the current study, several pro- and anti-apoptotic genes were differentially expressed at the 2 hour and 6 hour time points post-infection. These included *TNF* (upregulated, pro-apoptotic), the caspase 1, 4 and 6 genes (*CASP1CASP4CASP6*; all upregulated, all pro-apoptotic), the baculoviral IAP repeat containing 3 gene (*BIRC3*; upregulated, anti-apoptotic) and the CASP8 and FADD-like apoptosis regulator gene (*CFLAR*; upregulated, the isoforms of the protein encoded by this gene can act as either a pro- or anti-apoptotic mediators). Functional categorisation also revealed enrichment for genes involved in apoptosis at these two time points post-infection. The expression profiles of these genes concur with results from a recent study investigating the immuno-specific response of bovine MDM following 6 hpi with *M. avium* subsp. *paratuberculosis*[23].

While the upregulation of pro-apoptotic genes following *M. avium* subsp. *paratuberculosis* infection supports the induction of macrophage apoptosis, presumably to eliminate the intracellular pathogen [79], the upregulation of several anti-apoptotic genes suggests that this process is highly regulated. Anti-apoptotic signals may represent a host mechanism to limit the amount of host cell death following infection and may also enable improved antigen presentation by infected macrophages to T cells, leading either to elimination of the pathogen or control of infection in granulomas by adaptive immune mechanisms [80]. Alternatively, the induction of anti-apoptotic genes may signify immuno-subversion mediated by the pathogen, enabling survival and replication within the macrophage and evasion of the host immune response [81]. It is also possible that the pro- and anti-apoptotic signals detected here represent transcriptional signatures of “bystander” apoptosis, whereby uninfected macrophages undergo apoptosis following contact with mycobacteria-infected macrophages [23,82].

At the 6 hpi time point, there was a notable attenuation in the fold-change upregulation of many of the inflammatory cytokine genes (*e.g. CCL20*, *CXCL2*, *IL1B* and *TNF*) in the infected MDM compared with their fold-increase in expression at the 2 h time point. Furthermore, differential expression of *IL1A*, *IL6* and *IL10*―three cytokine genes each displaying > 3.6-fold induction 2 hpi―was no longer observed at the 6 h time point (although real time qRT-PCR data indicate that *IL6* was significantly upregulated at this time point (+2.51-fold, *P* ≤ 0.01)). In addition, genes encoding proteins involved in the MAPK cellular signalling pathway were not differentially expressed at this time point. These data suggest that the expression of key macrophage cytokines and chemokines that drive the inflammatory response to *M. avium* subsp. *paratuberculosis* infection, together with the MAPK cascade genes that encode signalling proteins regulating their expression, has largely waned 6 hpi.

It is possible that this attenuated fold-change in gene expression represents self-regulation of the host innate response to control the amount of cytokine and chemokine production during infection, thus limiting the amount of tissue damage caused during infection [83]. Interestingly, host cytokine-induced tissue damage is a mechanism that has been proposed to facilitate dissemination of mycobacterial infection [84]. However, pathogenic mycobacteria also have the ability to inhibit cytokine and chemokine production, such as inducing over-expression of anti-inflammatory cytokines including IL-10, which results in the abrogation of the host immune response and enables their persistence within infected macrophages [60]. Therefore, attenuation of the fold-increase in cytokine and chemokine expression 6 hpi compared with the fold-increase in expression of these genes at the 2 h time point, may reflect differential host- and pathogen-directed survival strategies during *M. avium* subsp. *paratuberculosis* infection.

Interestingly, two cytosolic PRRs―*TLR3* and *IFIH1*, whose proteins products are involved with the detection of viral PAMPs, such as reoviral double-stranded RNA―were upregulated at the 6 h time point post-infection. This observation is further supported by inspection of the differentially expressed genes within the IPA-identified *Role of PRRs in the detection of bacteria and viruses* pathway (4^th^ ranked pathway at the 6 h time point). TLR3 belongs to the TLR family of PRRs that localises to endosomes―membrane-bound vesicles inside eukaryotic cells that transport proteins and lipids from the cells surface and other organelles to the lysosome for degradation [68]. *IFIH1* encodes a cytosolic RIG-I-like receptor (RLR) RNA helicase that functions as cytoplasmic sensors of viral RNA [85,86].

The role of these cytosolic PRRs in mediating the macrophage response to *M. avium* subsp. *paratuberculosis* infection in vitro is intriguing given their documented roles in detecting viral PAMPs. One possibility for their induction following infection is that these PRRs mediate autophagy in infected macrophages―a complex cellular process in which host cell cytosolic components such as damaged or surplus organelles are engulfed in double-membrane vesicles, called autophagosomes, and fused with late endosomes or lysosomes to degrade their contents. Burgeoning evidence supports autophagy as a key host innate defence mechanism against mycobacterial infection, while cytosolic PRRs, including members of the TLR and RIG-I family of receptors, have been implicated in regulating this process [87-91]. Alternatively, it has been proposed that activation of cytosolic PRRs by intracellular bacterial pathogens may be due to the translocation of bacterial RNA into host cells, or is a consequence of the generation of host-derived RNA ligands caused by the pathogen-mediated disruption of host cellular pathways during infection [92].

There was a striking reduction in the number of differentially expressed genes at the 24 h time point compared to that for 2 and 6 hpi. Furthermore, the list of differentially expressed genes at this time point did not include any cytokine and chemokine genes. These data suggest that up- and down-regulation of macrophage genes in response to *M. avium* subsp. *paratuberculosis* infection has largely abated by the 24 h time point, supporting the hypothesis that changes in macrophage gene expression in response to *M. avium* subsp. *paratuberculosis* infection in vitro are transient [54,60]. However, among the differentially expressed genes identified 24 hpi with immunological function associated with mycobacterial infection was *CD40*; a macrophage receptor previously shown to be important for mediating the host response to mycobacterial pathogens.

CD40 encodes member 5 of the TNF-receptor (TNFR) superfamily of proteins, which is expressed on the surface of macrophages and other antigen presenting cells. Macrophage activation by antigen-activated T cells occurs through the binding of CD40 with the CD40 ligand (CD40L, which is transiently expressed on the surface of activated T cells) resulting in the induction of the genes encoding IL-12p40 (*IL12B*) and NOS (*NOS2*) [93]. Several studies have highlighted the importance of CD40 in the host response to mycobacterial infection. For example, Sommer et al. [94] demonstrated that bovine MDM infected with *M. avium* subsp. *paratuberculosis* did not induce *IL12B* and *NOS* expression following treatment with CD40L, suggesting that the pathogen suppresses *IL12* and *NOS* expression by interfering with CD40-mediated signalling. Also, CD40^−/−^ knockout mice infected with *M. avium* were shown to have impaired IL-12 and IFN-γ responses compared to control mice, suggesting that CD40 is important for the development of the adaptive immune response to mycobacterial infection [95].

In the current study, microarray analysis showed that *CD40* was upregulated at all time points post-infection, while *CD40 signalling* was the 7^th^ highest ranking canonical pathways identified 2 hpi, supporting an important role for this macrophage receptor during *M. avium* subsp. *paratuberculosis* infection in vitro. *IL12B* was differentially expressed (upregulated) in the infected MDM only at the 2 h time point, while *NOS2* was upregulated 2 and 6 hpi and was not differentially expressed at the 24 h time point. Thus, these findings lend some support to the hypothesis that *M. avium* subsp. *paratuberculosis* infection in vitro suppresses *IL12B* and *NOS2* expression in MDM via the CD40 signalling pathway 24 hpi [94].

In conclusion, the results from this study provide further insight in to the early transcriptomic macrophage response to *M. avium* subsp. *paratuberculosis* infection. In particular, inspection and systems analysis of the differentially expressed genes identified several key cellular pathways involved in the host response to *M. avium* subsp. *paratuberculosis*, including TLR, MAPK and CD40 signalling pathways, apoptosis and possibly autophagy. Further analyses involving the comparison of gene expression changes in bovine macrophages to other mycobacterial species, such as *M. bovis* and *M. bovis*-BCG, may also highlight the differential responses of the host macrophage to these pathogens and enable identification of key cellular pathways that contribute to the development of pathology. Furthermore, the identification of gene expression patterns linked to infection and pathology could provide a route to improved diagnostics of Johne’s disease.

## Misc

†These authors contributed to this work equally

## Competing interests

The authors declare that they have no competing interests.

## Authors’ contributions

Conceived and designed the experiments: DEM, MT, NCN, JAB, EG, DAM. Performed the experiments: DAM, MT, NCN, JAB. Analysed the data: DAM, MT, KEK, NCN, JAB, SDEP, KH. Wrote and edited the manuscript: DAM, DEM, MT, KEK, NCN, KH, EG. All authors read and approved the manuscript.

## Supplementary Material

Additional file 1 Table S1 The real time qRT-PCR primers used in this study. Click here for file

Additional file 2 Table S2 The list of differentially expressed transcripts detected at the 2 h time point post-infection. Click here for file

Additional file 3 Table S3 Gene ontology (GO) categories identified using IPA at the 2 h time point post-infection. The top ranking GO categories identified by IPA are listed according to *P*-values. Click here for file

Additional file 4 Table S4 Top-ranking canonical pathways identified using IPA at the 2 h time point post-infection. The top ranking canonical pathways identified by IPA are listed according to *P*-values. The ratio indicates the number of differentially expressed genes involved in each canonical pathway divided by the total number of genes within each pathway as per the IPA Knowledge Base. Click here for file

Additional file 5 Table S5 The list of differentially expressed transcripts detected at the at the 6 h time point post-infection. Click here for file

Additional file 6 Table S6 Gene ontology (GO) categories identified using IPA at the 6 h time point post-infection. The top ranking GO categories identified by IPA are listed according to *P*-values. Click here for file

Additional file 7 Table S7 Top-ranking canonical pathways identified using IPA at the 6 h time point post-infection. The top ranking canonical pathways identified by IPA are listed according to *P*-values. The ratio indicates the number of differentially expressed genes involved in each canonical pathway divided by the total number of genes within each pathway according to the IPA Knowledge Base. Click here for file

Additional file 8 Table S8 The list of differentially expressed transcripts detected at the at the 24 h time point post-infection. Click here for file

Additional file 9 Table S9 Gene ontology (GO) categories identified using IPA at the 24 h time point post-infection. The top ranking GO categories identified by IPA are listed according to *P*-values. Click here for file

Additional file 10 Table S10 Top-ranking canonical pathways identified using IPA at the 24 hour time point post-infection. The top ranking canonical pathways identified by IPA are listed according to *P*-values. The ratio indicates the number of differentially expressed genes involved in each canonical pathway divided by the total number of genes within each pathway according to the IPA Knowledge Base. Click here for file

Additional file 11 Table S11 Gene expression profiles for the *CCL5*, *CCL20* and *IL1B* genes based on real time qRT-PCR analysis using conventional and linearly amplified cDNA. Geometric mean fold-changes of gene expression (infected MDM relative to non-infected control MDM at each time point) are given for the microarray and real time qRT-PCR data at each time point. “NS” indicates that the gene was not significantly differentially expressed at the appropriate time point. Click here for file

Additional file 12 Figure S1 Comparison of the fold-changes in expression for the *CCL5*, *CCL20* and *IL1B* genes based on real time qRT-PCR analysis using conventional and linearly amplified cDNA. Log_2_ fold-changes in expression in the *M. avium* subsp. *paratuberculosis*-infected MDM (MPTb) relative to the non-infected control MDM at all three time points are shown. Linearly amplified cDNA template was prepared using the WT-Ovation™ RNA Amplification System (see Materials and Methods section). The significance of the fold-changes in expression for each gene based on the real time qRT-PCR analysis only are denoted by asterisks in the figure (*P ≤ 0.05, **P ≤ 0.01, ***P ≤ 0.001). In addition, the log_2_ fold-change in expression for the non-infected control MDM at each time point relative to the 0 hour non-infected control MDM are also shown for both the conventional and linearly amplified cDNA; no significant differences in gene expression between the non-infected control MDM relative to the 0 hour non-infected control was observed at each time point (*P* ≥ 0.05). Fitted line plots showing the regression analysis of the log_2_ fold-change in expression for the cDNA and linearly amplified cDNA are also presented. Click here for file

Additional file 13 Figure S2 Real time qRT-PCR analysis. Log_2_ fold-changes in expression in the infected MDM relative to the non-infected control MDM at all three time points are shown. Genes with an “OVA” suffix indicate that linearly amplified cDNA template was used for the analysis of these genes. For comparison, the expression profiles for these genes as per the microarray data are also shown. The significance of the mean fold-changes in expression for each gene based on the real time qRT-PCR analysis only are denoted by asterisks in the figure (*P ≤ 0.05, **P ≤ 0.01, ***P ≤ 0.001). The mean fold-changes calculated for each gene based on the microarray data in the infected MDM for each gene are detailed in Table 1. In addition, the log_2_ fold-change in expression for the non-infected control MDM at each time point relative to the 0 hour non-infected control MDM are also shown for both the microarray and real time qRT-PCR data (see the Results section of the manuscript detailing the comparison between the real time qRT-PCR and microarray results). Click here for file
