# Supplementary material for: Pan-genomic analysis of bovine monocyte-derived macrophage gene expression in response to in vitro infection with Mycobacterium avium subspecies paratuberculosis
Source: Vet Res. 2012 Mar 28;43(1):25. doi: 10.1186/1297-9716-43-25 (PMC3411445; doi:10.1186/1297-9716-43-25)
Supplement: Additional file 1 Table S1 — The real time qRT-PCR primers used in this study. [file 1297-9716-43-25-S1.doc]

**cDNA labelling protocol**

Total RNA was amplified using the NuGEN™ Ovation™ RNA Amplification System V2. First-strand synthesis of cDNA was performed using a unique first-strand DNA/RNA chimeric primer mix, resulting in cDNA/mRNA hybrid molecules. Following fragmentation of the mRNA component of the cDNA/mRNA molecules, second-strand synthesis was performed and double-stranded cDNA was formed with a unique DNA/RNA heteroduplex at one end. In the final amplification step, RNA within the heteroduplex was degraded using RNaseH, and replication of the resultant single-stranded cDNA was achieved through DNA/RNA chimeric primer binding and DNA polymerase enzymatic activity. The amplified single-stranded cDNA was purified for accurate quantitation of the cDNA and to ensure optimal performance during the fragmentation and labelling process. The single stranded cDNA was assessed using spectrophotometric methods in combination with the Agilent Bioanalyzer. The appropriate amount of amplified single-stranded cDNA was fragmented and labelled using the FL-Ovation™ cDNA Biotin Module V2. The enzymatically and chemically fragmented product (50-100 nt) was labelled via the attachment of biotinylated nucleotides onto the 3'-end of the fragmented cDNA.

**cDNA hybridisation protocol**

Fragmented and labelled cDNA was added to the hybridisation cocktail in accordance with the NuGEN™ guidelines for hybridisation onto Affymetrix GeneChip® arrays. Following the hybridisation for 16-18 hours at 45°C in an Affymetrix GeneChip® Hybridisation Oven 640, the array was washed and stained on the GeneChip® Fluidics Station 450 using the appropriate fluidics script, before being inserted into the Affymetrix autoloader carousel and scanned using the GeneChip® Scanner 3000 scan protocol. All Affymetrix Bovine Genome Array microarrays were scanned using an Affymetrix GeneChip® Scanner 3000.

**Microarray data processing**

Affymetrix® GeneChip® Bovine Genome Array data were analysed using Bioconductor [http://www.bioconductor.org] contained within the R statistical package (http://www.r-project.org). Normalisation of raw data was performed using the Factor Analysis for Robust Microarray Summarization (FARMS) algorithm. The FARMS algorithm uses only perfect match probes and a quantile normalization procedure, which provides the signal intensities.

**Value definition**

Normalized Log2 signal intensities
