# Supplementary figures and images for: Pan-genomic analysis of bovine monocyte-derived macrophage gene expression in response to in vitro infection with Mycobacterium avium subspecies paratuberculosis
Source: Vet Res. 2012 Mar 28;43(1):25. doi: 10.1186/1297-9716-43-25 (PMC3411445; doi:10.1186/1297-9716-43-25)

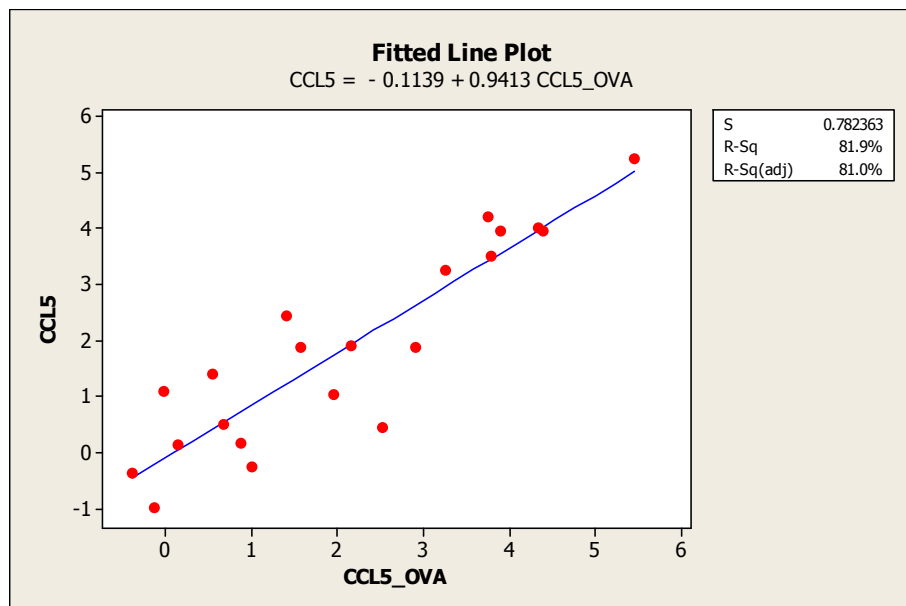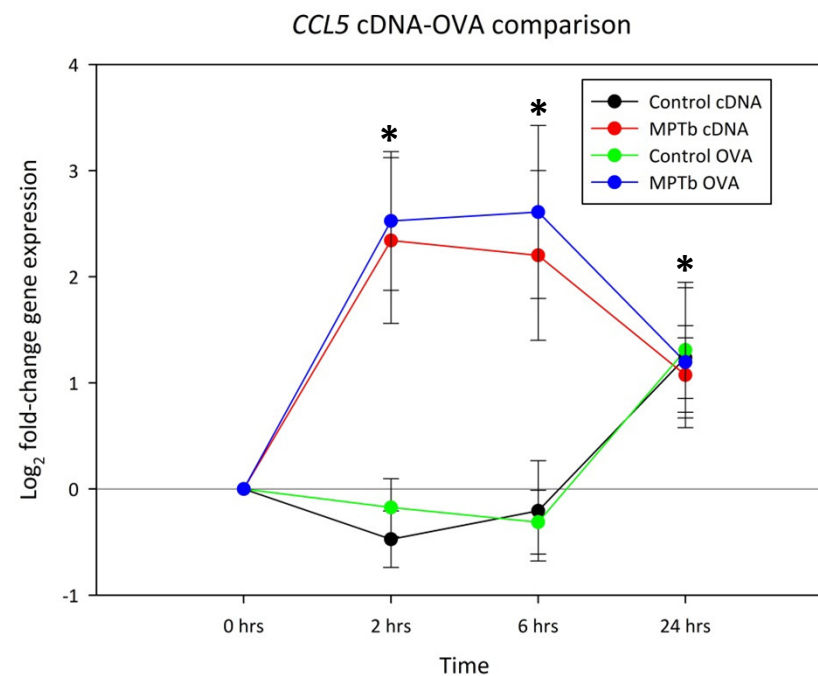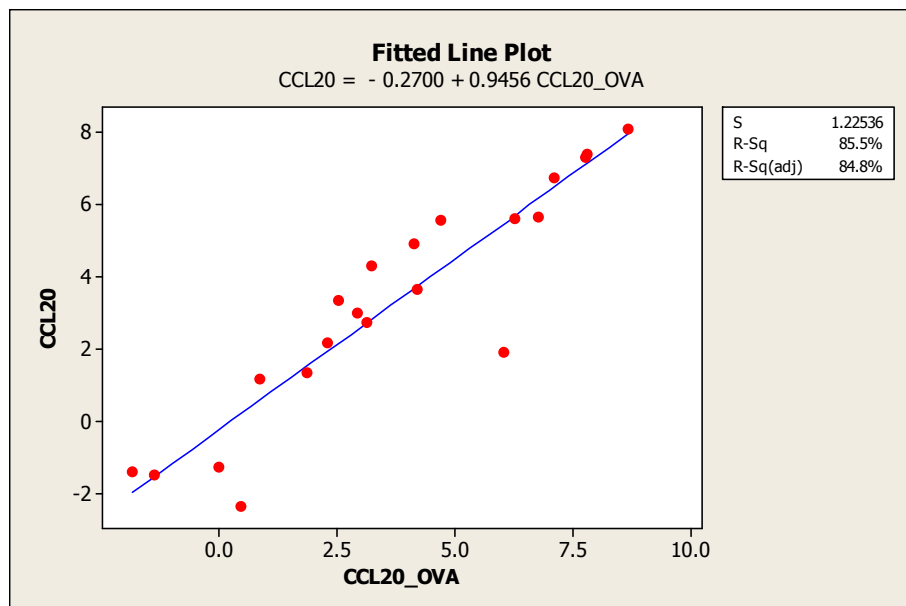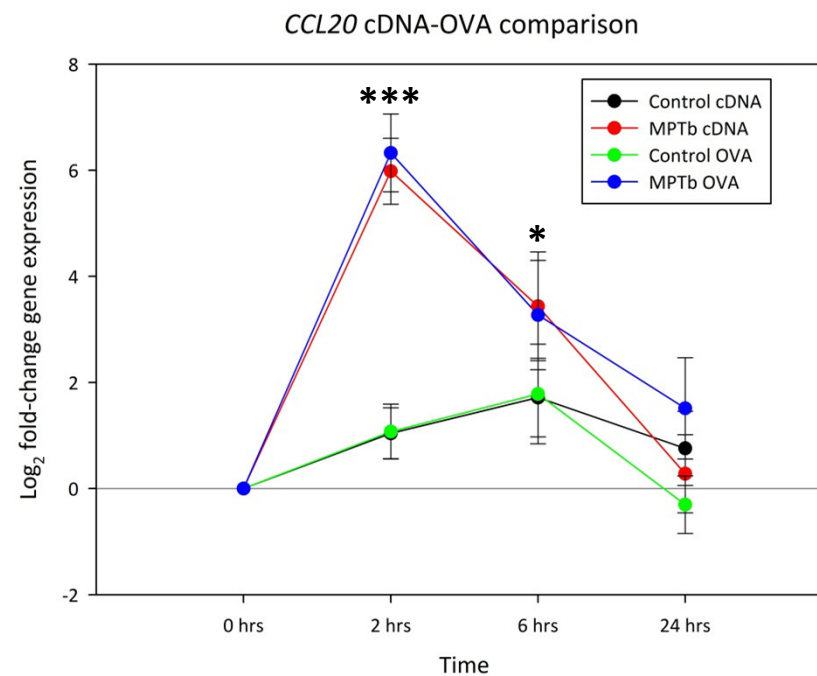

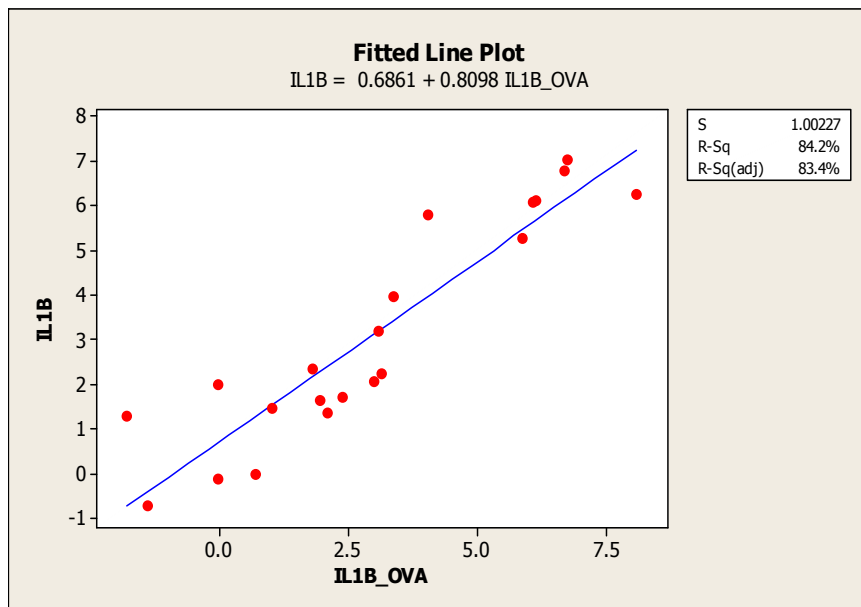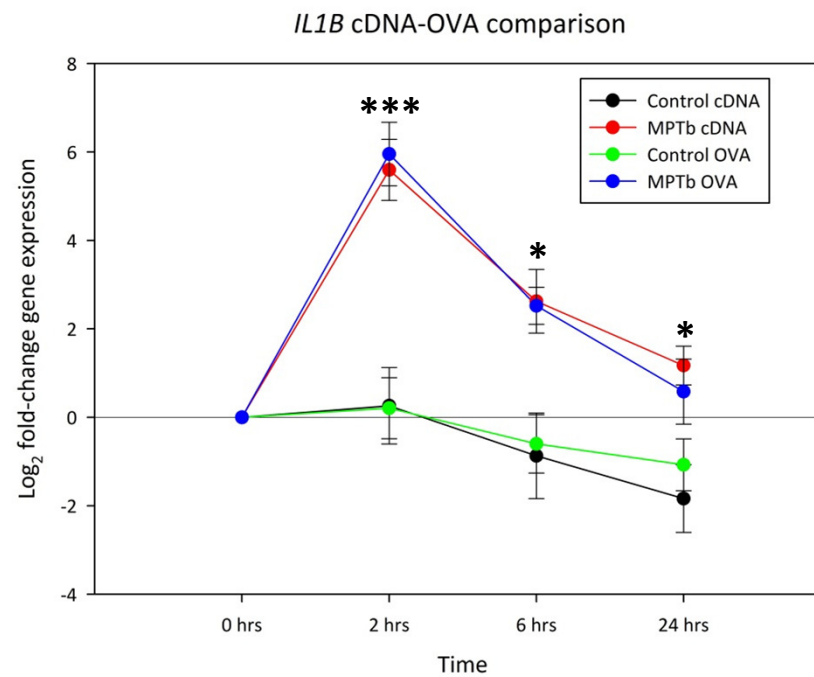

Supplement: Additional file 12 Figure S1 — Comparison of the fold-changes in expression for the CCL5, CCL20 and IL1B genes based on real time qRT-PCR analysis using conventional and linearly amplified cDNA. Log2 fold-changes in expression in the M. avium subsp. paratuberculosis-infected MDM (MPTb) relative to the non-infected control MDM at all three time points are shown. Linearly amplified cDNA template was prepared using the WT-Ovation™ RNA Amplification System (see Materials and Methods section). The significance of the fold-changes in expression for each gene based on the real time qRT-PCR analysis only are denoted by asterisks in the figure (*P ≤ 0.05, **P ≤ 0.01, ***P ≤ 0.001). In addition, the log2 fold-change in expression for the non-infected control MDM at each time point relative to the 0 hour non-infected control MDM are also shown for both the conventional and linearly amplified cDNA; no significant differences in gene expression between the non-infected control MDM relative to the 0 hour non-infected control was observed at each time point (P ≥ 0.05). Fitted line plots showing the regression analysis of the log2 fold-change in expression for the cDNA and linearly amplified cDNA are also presented. [file 1297-9716-43-25-S12.pdf]
