# Supplementary material for: Pan-genomic analysis of bovine monocyte-derived macrophage gene expression in response to in vitro infection with Mycobacterium avium subspecies paratuberculosis
Source: Vet Res. 2012 Mar 28;43(1):25. doi: 10.1186/1297-9716-43-25 (PMC3411445; doi:10.1186/1297-9716-43-25)
Supplement: Additional file 13 Figure S2 — Real time qRT-PCR analysis. Log2 fold-changes in expression in the infected MDM relative to the non-infected control MDM at all three time points are shown. Genes with an “OVA” suffix indicate that linearly amplified cDNA template was used for the analysis of these genes. For comparison, the expression profiles for these genes as per the microarray data are also shown. The significance of the mean fold-changes in expression for each gene based on the real time qRT-PCR analysis only are denoted by asterisks in the figure (*P ≤ 0.05, **P ≤ 0.01, ***P ≤ 0.001). The mean fold-changes calculated for each gene based on the microarray data in the infected MDM for each gene are detailed in Table 1. In addition, the log2 fold-change in expression for the non-infected control MDM at each time point relative to the 0 hour non-infected control MDM are also shown for both the microarray and real time qRT-PCR data (see the Results section of the manuscript detailing the comparison between the real time qRT-PCR and microarray results). [file 1297-9716-43-25-S13.doc]

**Table S11: Comparison of relative gene expression fold-changes in the MPTb-infected MDM obtained from real time qRT-PCR analysis using conventionally-prepared and linearly amplified cDNA.**

| **Time** | **2 hours** | | | | **6 hours** | | | | **24 hours** | | | |
| --- | --- | --- | --- | --- | --- | --- | --- | --- | --- | --- | --- | --- |
| **cDNA template** | **Conventional cDNA** | | **Amplified cDNA** | | **Conventional cDNA** | | **Amplified cDNA** | | **Conventional cDNA** | | **Amplified cDNA** | |
| **Gene** | **Mean fold-change** | ***P*-value** | **Mean fold-change** | ***P*-value** | **Mean fold-change** | ***P*-value** | **Mean fold-change** | ***P*-value** | **Mean fold-change** | ***P*-value** | **Mean fold-change** | ***P*-value** |
| *CCL5* | + 5.08 | ≤ 0.05 | + 5.78 | ≤ 0.01 | + 4.60 | ≤ 0.05 | + 6.11 | ≤ 0.05 | + 2.10 | ≤ 0.05 | + 2.29 | ≤ 0.05 |
| *CCL20* | + 63.19 | ≤ 0.001 | + 80.34 | ≤ 0.001 | + 10.82 | ≤ 0.05 | + 9.66 | ≤ 0.05 | + 1.22 | NS | + 2.85 | NS |
| *IL1B* | + 48.35 | ≤ 0.001 | + 61.97 | ≤ 0.01 | + 6.16 | ≤ 0.05 | + 5.74 | ≤ 0.05 | + 2.25 | ≤ 0.05 | + 1.50 | NS |
